# The Baltimore College of Dental Surgery

**Published:** 1870-11

**Authors:** 


### EDITORIAL DEPARTMENT.

---

*The Baltimore College of Dental Surgery.*—The *Thirty-first* annual session of this institution commenced on the 17th of Oct. last, with Infirmary practice and preliminary lectures. The corps of Dental Demonstrators has been increased to *five*, and an additional room fitted up for the Infirmary practice, in order that every facility may be afforded the student for acquiring practical knowledge. The Infirmary is now open during the entire year, the good effect of which course is apparent in a largely increased practice.

The number of patients applying each day for operations, is far in advance of other years, especially at the opening of the session.

The number of students in attendance is as large as at the same period last year, and in quality, gentlemanly conduct and application, would do honor to any institution in the land.

